# The role of sphingosine-1-phosphate in autophagy and related disorders

**DOI:** 10.1038/s41420-023-01681-x

**Published:** 2023-10-18

**Authors:** Siqi Xiao, Kaixin Peng, Congxin Li, Yuanyuan Long, Qin Yu

**Affiliations:** Department of Gastroenterology & Hepatology, Tongji Hospital, Tongji Medical College, Huazhong University of Science & Technology, Jiefang Avenue 1095#, Wuhan City, Hubei Province 430030 P.R. China

**Keywords:** Cell biology, Sphingolipids, Inflammatory bowel disease

## Abstract

S1P, also referred to as sphingosine-1-phosphate, is a lipid molecule with bioactive properties involved in numerous cellular processes such as cell growth, movement, programmed cell death, self-degradation, cell specialization, aging, and immune system reactions. Autophagy is a meticulously controlled mechanism in which cells repurpose their elements to maintain cellular balance. There are five stages in autophagy: initiation, nucleation, elongation and maturation, fusion, and degradation. New research has provided insight into the complex connection between S1P and autophagy, uncovering their interaction in both normal and abnormal circumstances. Gaining knowledge about the regulatory mechanism of S1P signaling on autophagy can offer a valuable understanding of its function in well-being and illness, potentially leading to innovative therapeutic concepts for diverse ailments. Hence, this review analyzes the essential stages in mammalian autophagy, with a specific emphasis on recent research exploring the control of each stage by S1P. Additionally, it sheds light on the roles of S1P-induced autophagy in various disorders.

## Facts

- Autophagy (Greek for “self-eating”) is a process of self-digestion, a fundamental cellular metabolic process closely related to health.
- S1P, a bioactive sphingolipid metabolite, has various roles in developmental, physiological, and pathological scenarios.
- Accumulating evidence indicates that S1P might be involved in the autophagy process.

## Open questions

- How does S1P regulate autophagy at the molecular level?
- How does S1P signaling affect autophagy in autophagy-related disorders?
- Considering the complex mechanism of S1P on autophagy regulation, how can we develop more precise targeted formulas for various autophagy-related diseases?

## Introduction

Sphingolipids, among the primary lipids in eukaryotes, are distinct from other lipids due to their utilization of sphingoid bases as building blocks [1]. In the past, sphingolipids were simply regarded as essential elements of cell membrane structure. Nevertheless, it is currently widely recognized that they possess biological activity and perform crucial functions in cellular processes like lipid metabolism and cellular signaling [2]. Ceramide, sphingosine, and S1P are the most studied bioactive sphingolipids. The synthesis, degradation, and interconversion of sphingolipids are regulated by a complex network of enzymes in sphingolipid metabolism. Ceramide plays a crucial role in the metabolism of sphingolipids, and S1P is produced through the consecutive enzymatic breakdown of ceramide [3].

Ceramide and S1P are believed to function as sphingolipid rheostats exhibiting antithetic properties in regulating various molecular mechanisms, including the mediation of the autophagy pathway [4]. Taniguchi et al. demonstrated that the sphingolipid rheostat controls autophagy and associated cell death via the mTOR pathway [5]. Previous studies have elaborated on the roles and mechanisms of ceramide in autophagy [6, 7]. Further systematic elucidation is required to understand the impact of S1P on autophagy. In recent times, an increasing number of research studies have indicated that S1P has the potential to impact autophagy, not just via the mTOR pathway [8–13]. This review provides an overview of the role of S1P in the fundamental mechanism of autophagy and elaborates on the influence of S1P on autophagy in various medical disorders.

## Overview of S1P

**Fig. 1:**
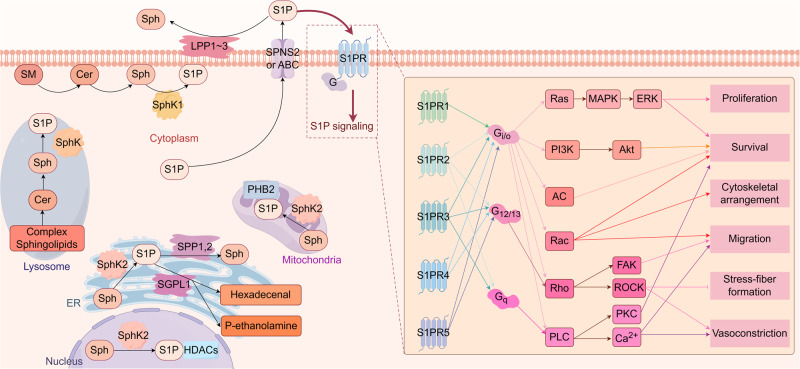
S1P metabolism and signaling. S1P is synthesized by SphK1 (mainly in the cytosol) and SphK2 (mainly in the mitochondria, ER, and nucleus). Intracellular S1P is degraded by SPPs and SGPL1 located in the ER, while extracellular S1P is degraded by LPPs located at the cell plasma membrane. S1P can be exported from cells through SPNS2, ABC transporters, or MFSD2B (only present in platelets and RBCs) and then acts on S1PRs to stimulate downstream signaling. S1P can also bind to intracellular targets, such as PHB2 (in the mitochondria) and HDACs (in the nucleus), to induce a series of signaling pathways. Created with figdraw.com. SM sphingomyelin, Cer ceramide, Sph sphingosine, SphK1,2 sphingosine kinases, SPP1,2 S1P phosphatases, SGPL1 S1P lyase, LPP1-3 lipid phosphate phosphatases, SPNS2 spinster homologue 2, ABC ATP-binding cassette transporter, MFSD2B major facilitator domain containing 2B, G G-protein, PHB2 prohibitin 2, HDACs histone deacetylases, ER endoplasmic reticulum.

The field of biomedical research has shown considerable interest in S1P. The general summary of S1P metabolism and signaling is shown in Fig. 1. S1P is synthesized by two sphingosine kinases, SphK1 and SphK2, which catalyze the phosphorylation of sphingosine to S1P. SphK1 primarily exists in the cytosol, while SphK2 is mainly located in the mitochondria, endoplasmic reticulum (ER), and nucleus [14]. SphK1 can translocate to the plasma membrane under stimulation, thereby facilitating the secretion of S1P [15]. Intracellular S1P can be transported to the extracellular domain through SPNS2, ABC transporters or MFSD2B (only present in platelets and RBCs) and then acts on different subtypes of S1P receptors (S1PRs) and initiates downstream signaling [16]. The levels of intracellular and extracellular S1P are determined by the equilibrium between synthesis and degradation. The degradation of intracellular S1P occurs in the ER: dephosphorylation into sphingosine through S1P phosphatases (SPPs) or irreversible cleavage into ethanolamine phosphate by S1P lyase (SGPL1) [17]. Extracellular S1P is mainly degraded via lipid phosphate phosphatases (LPPs) at the cell plasma membrane [18].

Extracellular S1P acts as a ligand for five GPCRs (S1PR1-5), which are extensively found in different tissues and cell types and are crucial in regulating diverse biological processes [19]. The spatial gradients of S1P allow selective receptor activation at multiple locations [20]. S1PRs are linked to various G-protein α subtypes, such as Gi/o, G12/13, and Gq, resulting in the control of distinct downstream signaling cascades and cellular responses [21]. Different cellular processes, such as cell proliferation, survival, migration, differentiation, and immune responses, are mediated by downstream signaling pathways activated by extracellular S1P through specific receptors [22–26]. Besides, intracellular S1P can serve as a second messenger and bind to intracellular targets. For example, S1P can bind with prohibitin-2 (PHB2) to affect mitochondrial respiration in the mitochondria [27] and combine with histone deacetylases to influence gene expression in the nucleus [28].

## Overview of autophagy

Autophagy, a cellular mechanism, is responsible for degrading and reusing damaged or unneeded cellular parts like proteins, organelles, and biomolecules to ensure cellular balance [29]. As a highly conserved process, autophagy occurs in every eukaryotic cell and plays a vital role in cell survival and adaptation. When subjected to stress, autophagy, in most cases, adaptively limits disorder and death [30]. Nevertheless, autophagy can promote cell apoptosis and necrosis in specific instances [31]. The disruption of autophagy has been linked to the development of different illnesses, such as cancer, neurodegeneration, and inflammatory diseases. Based on different substrate transport mechanisms to the lysosome, autophagy is categorized into three types: macroautophagy, microautophagy, and chaperone-mediated autophagy [32]. This review focuses on macroautophagy (hereafter referred to as autophagy), which stands out as the foremost and extensively researched type.

**Fig. 2:**
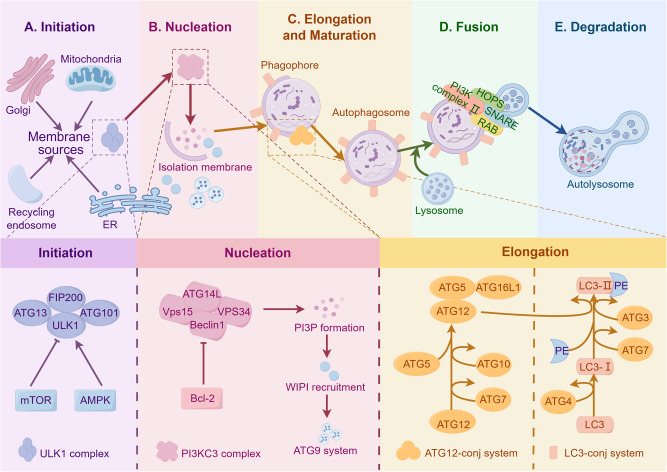
Molecular mechanism of autophagy. The process of autophagy at the molecular level involves five steps: initiation, nucleation, elongation and maturation, fusion, and degradation. The ULK1 complex (including ULK1, FIP200, ATG13, and ATG101) is the primary initiator of autophagy and can be regulated by mTOR and AMPK. The ULK1 complex activates the critical factor of nucleation, PI3KC3 complex (including Beclin1, ATG14L, Vps15, and Vps34), and recruits downstream proteins, such as WIPI proteins, ATG9-positive vesicles and ATG5-ATG12-ATG16L1 complex, for the elongation and maturation. LC3-I converts to LC3-II to facilitate autophagosome maturation. PI3K complex II, HOPS complex, RAB proteins, SNARE proteins, and the LC3 family proteins function in the fusion. Finally, the engulfed cargo is degraded by lysosomal hydrolases and produces small molecules recycled to the cytosol. Created with figdraw.com. PI3P phosphatidylinositol 3-phosphate, PE phosphatidylethanolamine, HOPS homotypic fusion and vacuole protein sorting, RAB targeting GTPase, SNARE soluble N-ethylmaleimide-sensitive factor attachment protein receptor.

Autophagy occurs by forming a dual-layered vesicle known as the autophagosome, which envelops the cytoplasmic contents to be broken down. Afterwards, the autophagosome combines with lysosomes, resulting in the formation of the autolysosome, where the load is degraded by lysosomal hydrolases. Finally, the resultant breakdown products are released back into the cytoplasm for reuse, establishing a dynamic recycling system [33]. At the molecular level, autophagy can be categorized into five stages (Fig. 2): initiation of autophagy, nucleation of vesicles (formation of the isolation membrane/phagophore), elongation and maturation of vesicles, fusion of the autophagosome with lysosome, and degradation of contents in the package [34]. The regulation of autophagy involves an intricate system of signaling pathways, such as the mammalian target of rapamycin (mTOR) pathway and the AMP-activated protein kinase (AMPK) pathway, which are well-known triggers of autophagy and closely control the inhibition and activation of the key initiator of autophagy, the Unc-51-like kinase 1 (ULK1) kinase complex [35]. The activation of the ULK1 complex, which consists of ULK1, FIP200, ATG13, and ATG101, stimulates the class III PI3K (PI3KC3) core complex and a series of autophagy cascade reactions [36]. PI3KC3 complex, which contains Beclin1, ATG14L, Vps15, and Vps34, then produces a phosphatidylinositol 3-phosphate (PI3P) pool and recruits downstream proteins, such as WIPI proteins, ATG9-positive vesicles and ATG5-ATG12-ATG16L1 complex, to form phagocytic vesicles. During the elongation and closure of the autophagosome membrane, the LC3 family proteins modified with phosphatidylethanolamine (PE) can bind to autophagic receptors and promote autophagosome maturation [37]. Next, the autophagosome fuses with lysosome or late endosome, and the fusion requires the assistance of UVRAG-containing class III PI3K complex (PI3K complex II), HOPS complex, RAB proteins, SNARE proteins, and the LC3 family proteins [38]. Eventually, the engulfed cargo undergoes degradation, and the resultant tiny compounds can be reused within the cytosol.

## Regulation of autophagy by S1P

### Autophagosome initiation

**Fig. 3:**
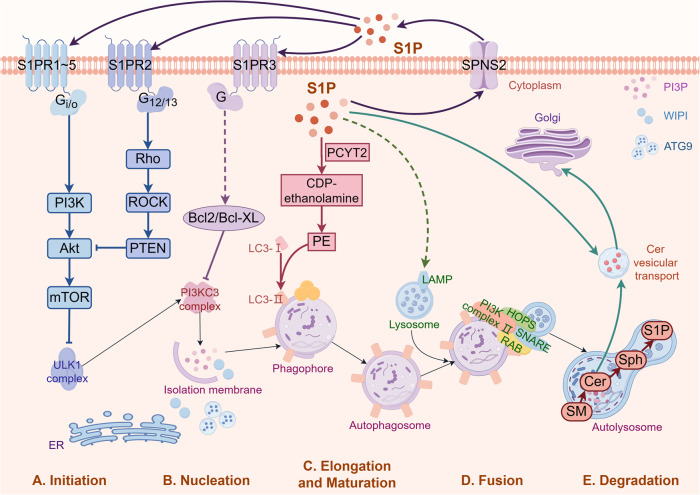
Regulatory role of S1P in the core process of autophagy. S1P is synthesized in the cell and transported to the extracellular space through SPNS2 or ABC. By coupling with Gi/o, S1P/S1PRs activate the PI3K/Akt/mTOR pathway. mTOR signaling phosphorylates the ULK1 complex to inhibit autophagy initiation. Besides, S1PR2 activates the G (12/13) /Rho/Rho kinase/PTEN pathway and leads to Akt inhibition. During vesicle nucleation, S1P/S1PR3 signaling upregulates the expression and phosphorylation of Bcl-2. The binding of Bcl-2 to Beclin1 helps Beclin1 escape from the PI3KC3 complex, leading to the failure of recruiting other autophagic proteins. PE functions as an anchor to phagophore membranes for LC3 to maintain vesicle elongation and maturation. Through directly interacting with S1P degradation product CDP-ethanolamine, PE connects S1P metabolism and autophagy regulation. The intracellular accumulation of S1P participates in the control of lysosome function by regulating lysosome calcium storage and the activity of lysosome-related membrane protein LAMP, thus regulating autophagosome-lysosome fusion. Finally, the fusion of autophagosome and lysosome provides a membrane source of SM for the production of S1P. Created with figdraw.com. PCYT2 ethanolamine, LAMP lysosome-associated membrane protein.

The mTOR signaling pathway is a central regulator of autophagy. It has a multifaceted impact on the initiation and subsequent stages of the autophagy process, as has been extensively documented [39, 40]. By directly phosphorylating and suppressing the ULK1 complex, which is crucial for the initiation of autophagy, mTOR hinders the process of autophagy [41]. Autophagy initiation can be regulated by S1P through modulation of mTOR signaling, as indicated by recent research (Fig. 3). In particular, S1P/S1PRs signaling has been demonstrated to stimulate mTOR and subsequent signaling pathways (Table 1).

**Table 1:** S1P/S1PRs-mTOR pathway regulates cellular events.

S1PRs	Pathway	Effect	Event	Reference
S1PR1	PI3K/Akt	mTOR activation	directs the reciprocal differentiation of Th1 and Treg cells	[50]
			antagonizes Treg cell-mediated immune suppression	[51]
			controls the development, maintenance and suppressive activity of natural regulatory T cells	[52]
			participates in immune regulation during bone remodeling	[53]
			differentially regulates the allogeneic response of CD4 and CD8 T cells by modulating mitochondrial fission	[54]
			induces rapid mobilization and high proliferation of HSPCs	[55]
			associates with the development of AD-like pathology	[56]
			plays an important role in neuronal growth and survival	[57]
			attenuates apoptosis of endothelial progenitor cells	[58]
			affects glucose metabolism (S1PR1-3)	[62]
S1PR2	PI3K/Akt	mTOR activation	impairs autophagy in astrocyte and microglia	[12, 65]
			affects glucose metabolism	[71]
			protect HUVECs from injury and inflammation	[70]
			promotes the proliferation of prostate cancer cells	[69]
			regulates cardiomyocyte survival from myocardial ischemia-reperfusion (I/R) injury	[73]
			involves in the pathogenesis of hepatocellular steatosis (S1PR2-3)	[59]
	Rho/Rho kinase/PTEN	mTOR inhibition	attenuates vascular barrier disruption	[67]
			inhibit cell migration	[66]
S1PR3	PI3K/Akt	mTOR activation	exacerbates lung cancer-associated inflammation	[60]
			Regulates HIF-1 in thyroid follicular carcinoma cells	[61]
			modulates autophagy and its associated cell death	[5]
S1PR4	/	mTOR activation	enhances the energy load of astrocytes	[65]
S1PR5	PI3K/Akt	mTOR activation	affects apoptosis and autophagy in MM cells	[47]

*HSPCs* hematopoietic stem and progenitor cells, *HUVECs* human umbilical vein endothelial cells, *HIF-1* hypoxia-induced factor-1, *MM* multiple myeloma.

Through downstream signaling molecules, extracellular S1P tightly binds to S1PR1-5 with a strong affinity, acting as an autocrine or paracrine signal to control cell behavior. S1P/S1PR/G-proteins have the ability to trigger different signaling pathways within the cell, such as PI3K/Akt/mTOR [42]. Previous research indicates that Akt serves as a mechanical connection between S1P and mTOR signaling [43], which can be activated by all Gi/o-coupled S1PRs, including S1PR1-5 [21, 44–47].

PI3K and Akt activation through Gi/o is primarily mediated by S1PR1 and S1PR3 [48, 49]. PI3K, Akt, and mTOR activation by S1P/S1PR1 have been demonstrated to control multiple cellular processes in the immune system, nervous system, and the proliferation of endothelial progenitor cells [50–58]. Activation of the Akt/mTOR pathway through S1P/S1PR3 signaling can regulate inflammatory factors, tumor regulators, and steatosis [59–61]. The activation of the Akt/mTOR pathway through S1PR1/3 signaling results in the inhibition of autophagy [5, 62, 63]. Notably, Liu et al. found that S1PR1 can activate mTOR through Akt-independent mechanisms, which warrants further investigation [64].

In an mTOR-dependent manner, S1P additionally functions as a ligand for S1PR2 to impact autophagy [12, 62, 65]. However, S1PR2 ligation probably leads to Akt inhibition [48]. According to reports, S1PR2 plays an active role in controlling the G (12/13) /Rho/Rho kinase/PTEN pathway, thereby suppressing Akt signaling [66–68]. In certain circumstances, S1PR2 can activate mTOR by enhancing the PI3K-Akt signaling [59, 69–73], potentially due to decreased receptor expression [74].

Despite the limited number of studies on the associated roles of S1PR4 and S1PR5, it has been reported that S1P/S1PR4 & 5 signaling can cause mTOR activation [47, 65, 75]. The regulation of S1P/mTOR on autophagy is a complex and dynamic process. Although the majority of research indicates that S1P suppresses autophagy by targeting mTOR, findings suggest that S1P can also trigger autophagy by inhibiting mTOR during nutritional deprivation [76, 77].

### Membrane nucleation and phagophore formation

The nucleation depends on the presence of the PI3KC3 complex, allowing the enlistment of additional proteins [78, 79]. The core compound of the PI3KC3 complex, Beclin1, facilitates the activation of Vps34 and the localization of different autophagy-related proteins to the pre-autophagosomal membrane [80–82]. Hence, the increase in Beclin1 can indicate the accumulation of autophagosomes under certain circumstances.

According to reports, the maintenance of autophagy necessitates the interaction of Beclin1 with the anti-apoptotic protein Bcl-2 [83]. Studies have demonstrated that S1P increases the expression of Bcl-2 in different cell types, such as myocytes, endothelial cells, primary macrophages, fibroblasts, keratinocytes, and liver sinusoidal endothelial cells [8, 84–88]. The upregulation of Bcl-2 promotes cell survival and prevents apoptosis. From Potteck et al., S1P/S1PR3 signaling might be involved in the anti-apoptotic process by enhancing the phosphorylation of Bcl-2 and altering the mitochondrial membrane potential [89]. Beclin1 binds to Bcl-2 and exits the Vps34/Vps15 complex in the presence of nutrient deficiency, resulting in membrane nucleation interruption and autophagy inhibition [30, 83, 90]. However, in certain instances, S1P can increase the expression of Beclin1, leading to the augmentation of pre-autophagosomal Beclin1-positive structures and the formation of autophagosome membranes [8, 10, 11].

### Phagophore expansion

Microtubule-associated protein light chain 3 (LC3) is a crucial protein in the control of autophagy, serving as a distinct indicator for autophagic vesicles [91]. There are two forms of LC3, namely LC3-I and LC3-II, where LC3-I is found in the cytoplasm, and LC3-II is found bound to the membrane [92]. During the elongation stage, LC3-I converts into LC3-II (PE-bound form) and moves to the autophagosome membrane, an essential process in autophagosome maturation [93]. Research has shown that S1P can boost the ratio of LC3 II/I and promote the formation of LC3-positive autophagosomes [76, 92]. This ratio is increased by the overexpression of SphK1 and decreased under the application of the S1P analog FTY720 [30, 77].

The influence of S1P on LC3 could be connected to the impact of ethanolamine phosphate, a degradation byproduct of S1P, which can be transformed into CDP-ethanolamine through ethanolamine. CDP-ethanolamine is subsequently incorporated into PE [94]. PE acts as a tether for phagophore membranes, securing LC3 and attaching LC3-I to phagophore membranes as LC3-II [95, 96]. The attachment of LC3 to the phagophore membrane facilitates the enlistment of cargo and the elongation of the phagophore membrane [97]. Therefore, PE establishes the connection between S1P metabolism and autophagy regulation (Fig. 3).

### Fusion

S1P has also been shown to regulate autophagy by modulating autophagosome-lysosome fusion (Fig. 3). Autophagic vesicles merge with endosomes or lysosomes after engulfing cytoplasmic cargo, resulting in the formation of autolysosomes [98–100]. As a result, autophagic activity is linked to lysosomal integrity.

S1P has been reported to promote autophagy-linked lysosomal degradation [101, 102]. Specifically, S1P plays a role in membrane transport, thus protecting the lysosomal structure and controlling lysosomal homeostasis [103, 104]. Lack of S1P hampers this homeostasis, consequently impeding the merging of autophagosomes with lysosomes, resulting in fatal autophagy [105]. According to reports, calcium in lysosomes can regulate the autophagic pathway at different levels [106]. The enhancement of lysosomal calcium storage can be influenced by S1P, which may be associated with the activation of the calcium-dependent mechanism of lysosomal maturation [101, 107]. Additionally, S1P has been demonstrated to regulate the activity of lysosome-associated membrane proteins (LAMPs). According to reports, the accumulation of intracellular S1P can destroy the function of lysosomes by influencing the levels and activity of LAMPs [108, 109]. Hence, a defined amount of S1P is necessary to maintain the function of autolysosomes, ensuring the efficient breakdown of cargo and the successful recycling of its constituents.

### Selectivity of autophagy

The type of degraded cargo determines whether autophagy is selective or non-selective. Lysosomes break down specific cellular parts, such as impaired or excess organelles, proteins, and pathogens, through a mechanism called selective autophagy [110, 111]. Selective autophagy can be categorized into different types including mitophagy, pexophagy, lipophagy, glycophagy, ribophagy, ER-phagy, and xenophagy, based on the cargo types [112]. In contrast to the overall degradation of packaged goods in non-selective autophagy, selective autophagy involves the initial ubiquitination labeling of specific cargo [113]. Recent studies have suggested that S1P may have a role in controlling selective autophagy (including mitophagy and pexophagy), emphasizing the intricacy of S1P signaling in regulating autophagy and its potential influence on disease pathogenesis [48].

Mitophagy, the most well-known form of selective autophagy that aims to degrade damaged mitochondria, is associated with S1P signaling [114]. The utmost importance for mitophagy is to maintain cellular homeostasis and prevent the accumulation of dysfunctional mitochondria [115]. The disruption of mitophagy has been linked to various neurodegenerative disorders, such as Parkinson’s and Alzheimer’s disease. Research has indicated that the activation of S1P signaling triggers the production of multiple mitophagy-related proteins, such as Bnip3l, Pink1, and p62, thereby facilitating the process of mitophagy [116, 117]. Furthermore, it has been reported that S1P regulates mitochondrial respiration by interacting with PHB2, a mitophagy protein with high affinity and specificity that regulates mitochondrial function and presents at the inner mitochondrial membrane [118, 119]. Through the function of PHB2, S1P can recruit LC3 on autophagosomes to regulate mitophagy [120].

Additionally, S1P has been documented to participate in the cellular mechanism of xenophagy. Xenophagy aims to degrade invading pathogens and support the immune system of the body [113]. In contrast to other forms of selective autophagy that target cellular components, xenophagy specifically targets intruders that oppose host cells [121]. By utilizing their functional proteins, several pathogens can evade the host immune response and prolong their survival within the host. For example, Legionella pneumophila can delay its delivery to lysosomes and interfere with the host autophagy. Rolando et al. discovered that S1P plays a role in the evasion process of L. pneumophila via a protein encoded by Lactobacillus pneumoniae, LpSPL, which is closely homologous to SGPL1 [122]. The control of sphingolipids degradation by LpSPL in host cells restricts autophagy while infected with L. pneumophila.

### Autophagy regulates S1P metabolism

Up to this point, the majority of research has shown the controlling impact of S1P on autophagy, yet there is a scarcity of literature reporting the control of S1P by autophagy. Practically speaking, autophagy may have a regulatory impact on the metabolism of S1P. The findings from Harlé et al. reveal that inflammation-induced autophagy in lymphatic endothelial cells (LECs) facilitates the breakdown of SphK1, resulting in a reduction in S1P synthesis [123]. Since SphK1 displays a high PSSM score of the LC3 interaction motif, they predict its effective autophagosome targeting, which needs further investigation. In human macrophages, SphK2 is reported to have no interaction with LC3 and might undergo degradation through LC3-independent alternative autophagy [124].

## S1P-mediated autophagy regulation in diseases

### Cancer

The well-documented significance of S1P in cancer development and progression encompasses its impact on cell survival, proliferation, migration, inflammation, angiogenesis, and the enlistment of immune cells [125–127]. Autophagy plays an intricate and situation-dependent role in cancer as a type II programmed cell death [128, 129]. Emerging data indicate that S1P can influence the development and spread of tumors through its regulation of autophagy in tumor cells.

Research has indicated that autophagy has a dual function in the regulation of cell death [130]. Autophagy typically exhibits its suppressive impact on tumors during the initial phase of tumor development. SphK1/S1P/S1PR3 signaling is activated in Lymphangioleiomyomatosis (LAM), leading to mTORC1 activation and autophagy inhibition [131]. The inhibition of S1P signaling has been verified to trigger autophagy-mediated cell death, resulting in decreased viability, migration and invasion of cancer cells. Insufficient intracellular S1P in multiple myeloma (MM) cells also reduces cell viability via autophagy [132]. Lima et al. investigated the correlation between the tumor suppressor TP53 and SphK1 in the regulation of autophagy [133]. They unveiled that the inhibition of SphK1 enhanced autophagy in a TP53-dependent manner, thus suppressing cancer cell growth and clonogenic survival. There have been reports indicating that selective blockers of SphK2 induce autophagy in tumor cells of kidney, prostate, breast, and T-cell acute lymphoblastic leukemia, ultimately resulting in nonapoptotic cell death and delayed tumor development [134–136]. Furthermore, the non-selective S1PR modulator fingolimod (FTY720), an analog of S1P, has shown the ability to trigger autophagic cell death and apoptosis in multiple myeloma (MM) cells [137, 138]. Considering the aforementioned discoveries, we speculate that obstructing S1P signaling during the initial phase of tumor formation to induce deadly autophagy could serve as a hopeful approach to hinder cancer growth.

As a cellular protective, survival, and defense mechanism, autophagy can also promote tumor growth under certain circumstances, especially in the late stage of tumor progress. Stressors acting on cancer cells, such as nutrient starvation, can activate SphK activity and autophagy, thus ensuring cell survival and resistance [139]. Numerous studies indicate that S1P induces protective autophagy in tumor cells. In human prostate cancer PC-3 cells, extracellular S1P is confirmed to activate S1PR5 to trigger cytoprotective autophagy by generating ER stress [13, 76]. Moreover, in human breast cancer MCF-7 cells, the upregulation of SphK1 or the suppression of SPP1 can enhance the levels of intracellular S1P and induce pro-survival autophagy [77, 140, 141]. In hepatocellular carcinoma (HCC) and colon cancer, the irreversible breakdown of S1P by SGPL1 triggers aberrant S1P signaling, resulting in the suppression of autophagy and the development of cancer [142]. According to the proposal of Wang et al., SphK1/S1P-induced autophagy can enhance the survival of cancer cells, making the inhibition of SphK1 a new anti-cancer strategy [143]. Notably, S1P analog FTY720 has been found to trigger protective autophagy in cancer cells, such as ovarian cancer cells, acute lymphoblastic leukemia cells, and melanoma cells [144–146]. This phenomenon could be attributed to the highly cell-type dependent effect of FTY720 on autophagy activation. Furthermore, autophagy can enhance the malignancy of tumors by promoting metastasis [130]. The increase of intracellular S1P in HCC tissue is reported to facilitate this process by activating TNF receptor-related factor 2 (TRAF2) [101]. Besides, excessive autophagy has been shown to interfere with S1P production and signaling, consequently amplifying the invasiveness of oncogenic K-Ras cells [147]. This phenomenon suggests a mutual influence between S1P and autophagy in the promotion of cancer metastasis.

### Nervous system diseases

The nervous system is a highly complex and essential network within the human body. It has a crucial function in coordinating various physiological and behavioral processes. Malfunction of the nervous system can appear in various neurological conditions, such as Alzheimer’s disease, Parkinson’s disease, multiple sclerosis, and Huntington’s disease (HD). As a crucial modulator of mammalian neural integrity, dysregulation of autophagy can induce the accumulation of multifunctional proteins and organelles, ultimately undermining the pro-survival and anti-apoptosis functions of autophagy in neurons [148, 149]. By controlling the different stages of autophagy in neurons and glial cells, S1P can promote the development of diverse neurological disorders.

It is reported that the increase of extracellular S1P boosts the energy burden on astrocytes via S1PR2/4 in either an autocrine or paracrine fashion, consequently exerting a detrimental impact on astrocytic autophagy [65]. Mitroi et al. found that SGPL1 deficiency in the brain can affect the neuronal health of mice [150]. They argue that the absence of SGPL1 leads to the buildup of S1P, which binds to S1PR2 and inhibits neuronal autophagy in an mTOR-dependent manner, resulting in the accumulation of easily aggregated proteins [12, 96, 151]. Furthermore, in a rat autism model, blocking SphK2/S1P signaling can enhance the expression of autophagy-related proteins and partially safeguard hippocampal neurons against deterioration [152]. Huntingtin is a protein that induces neurotoxicity and causes HD. Moruno et al. demonstrated that enhanced autophagy in neurons facilitates the breakdown of mutant huntingtin, thus exerting a pro-survival effect in HD [10]. During this process, the autophagic flux is enhanced by SphK1 and reduced by the S1P metabolic enzymes SGPL1 and SPPs. And S1P may coordinate the ER-dependent biogenesis of neuronal autophagosomes.

Although autophagy is mainly neuroprotective, it can induce excessive autophagy or lysosomal instability in specific pathological situations, contributing to neuronal loss in neurodegenerative disorders [153]. FTY720 is reported to have a robust neuroprotective effect on stroke [154]. Given the evidence that SphK1/S1P triggers autophagy and causes neuronal harm to microglia during brain ischemia-reperfusion through a TRAF2-dependent mechanism [155], it is logical to assume that the stroke-protective effect of FTY720 involves the inhibition of neuronal autophagy via the S1P signaling pathway [30].

### Cardiovascular diseases

In cardiovascular diseases, autophagy is crucial in protecting cardiomyocytes from stress-induced damage, including ischemic death and myocardial infarction (MI) [29, 156]. Besides, S1P/S1PR1 signaling has garnered significant interest due to its numerous effects on vascular and myocardial cells [157]. Therefore, the role of S1P in regulating autophagy in cardiovascular disorders has been widely investigated in recent times. The inhibition of mTOR by S1P is reported to induce myocyte autophagy, which plays a role in attenuates left ventricular remodeling and dysfunction following MI [8].

However, when autophagy is strongly induced, it can damage the cardiovascular system [158]. For instance, in models of stress-induced cardiomyopathy, there is a notable rise in cardiac autophagy, which can be subsequently reduced following S1P treatment [159]. The downregulation of S1PR1 in vivo has been demonstrated to trigger autophagy and impair cardiac function. Furthermore, in the context of load-induced heart failure, excessive autophagy results in autophagic cell demise and the depletion of cardiomyocytes [160]. According to reports, S1P/S1PR signaling can hinder the excessive autophagy of cardiomyocytes, thereby improving heart function during heart failure [161]. Interestingly, individuals diagnosed with pulmonary arterial hypertension (PAH) exhibit a rise in S1P levels. Studies have shown that S1P triggers autophagy by TRAF2-induced BECN1 expression and ubiquitination, thereby promoting the proliferation of pulmonary artery smooth muscle cells [11]. Hence, the prevention and treatment of PAH can be achieved by the blockage of S1P to inhibit autophagy.

### Respiratory diseases

According to recent research, autophagy in respiratory diseases, such as chronic obstructive pulmonary disease (COPD), pulmonary fibrosis, and asthma, is regulated by S1P signaling. It has been determined that COPD lungs exhibit increased autophagy. The impact of S1P on the autophagy of human lung microvascular endothelial cells (HLMVECs) was examined by researchers [102]. The researchers discovered that the rise in S1P levels triggers baseline autophagy in HLMVECs, a process that is inseparable from the function of S1PR1. Compared to non-smokers, the expression of S1PR1 in HLMVECs is notably reduced in smokers, leading to the progress of COPD. Huang et al. proposed that S1P signaling plays a role in the proliferation and differentiation of lung fibroblasts by regulating autophagy, thereby controlling the progression of pulmonary fibrosis [162]. SGPL1 can potentially serve as a new endogenous inhibitor of pulmonary fibrosis by modulating S1P signaling and autophagy [163]. S1P is additionally implicated in the inflammation generation and airway restructuring in asthma, potentially linked to the activation of S1PR2 and autophagy stimulation via the RAC1/JNK pathway [9]. In addition, S1P/S1PR3 signaling can participate in sepsis prevention by activating ERK1/2 and p38 to prevent excessive autophagy and stabilize pulmonary epithelial barrier integrity [164]. Modifying S1P signaling in immune cells such as T cells and macrophages could offer therapeutic advantages for respiratory disorders.

### Digestive system diseases

Autophagy disorders have been implicated in the pathogenesis of various digestive diseases, including pancreatitis, IBD, colon cancer, and liver diseases [165]. S1P receptors are widely expressed in the digestive system and play different roles in autophagy regulation. High SGPL1 levels in HCC and colon cancer inhibit protective autophagy, thereby promoting cancer development (as described in the *Cancer* section). During chronic pancreatitis, both plasma and pancreas exhibit elevated S1P levels. Studies indicate that the activation of pancreatic stellate cells and the development of pancreatic fibrosis are facilitated by S1P/S1PR2 signaling, which regulates autophagy and the NLRP3 inflammasome sequentially [166]. There are currently no studies reporting the direct effect of S1P on autophagy in IBD. Based on our previous research, we speculate that S1P could affect the development of IBD through autophagy regulation.

**Fig. 4:**
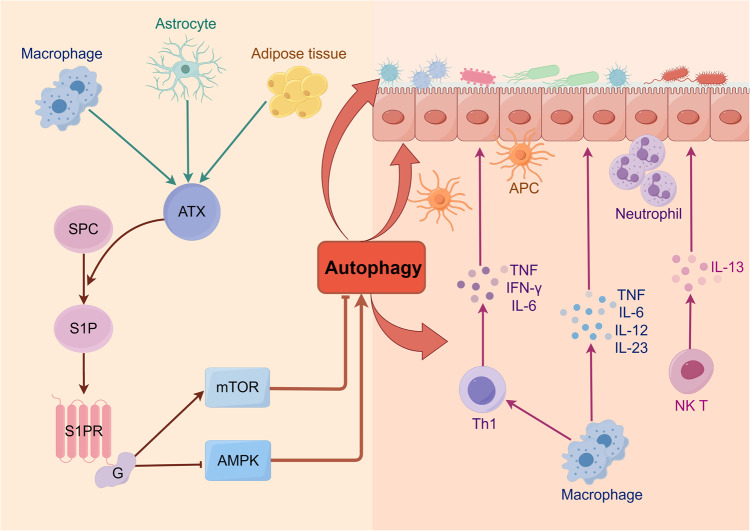
Possible regulation of S1P on autophagy in IBD. ATX is mainly derived from adipose tissue and can also be secreted by other cells, such as inflammatory macrophages and activated astrocytes. ATX can act as a synthase of S1P, degrading SPC to S1P. An increase in the level of ATX was detected in IBD. We suspect that ATX induces the S1P/S1PR/mTOR pathway and inhibits the S1P/S1PR/AMPK pathway to inhibit autophagy. The dysregulated autophagy then affects the distribution of gut microbiota, the immune response of intestinal mucosa, and the expression of tight junction protein, thereby affecting the epithelial barrier function, leading to the occurrence and development of IBD inflammation. Created with figdraw.com. ATX autotaxin, SPC sphingosylphosphorylcholine, APC antigen presenting cells, NK T natural killer T cells.

IBD, a chronic relapsing non-specific intestinal inflammatory disease, is believed to be influenced by various factors, including genetic background, environmental and luminal factors, and mucosal immune disorders [167, 168]. Certain types of IBD have been linked to genetic mutations in autophagy-related genes (ATG) [169, 170]. Studies have discovered that autophagy dysregulation can alter the intestinal microbiota composition, impact immune response, and disrupt intestinal barrier function, thus amplifying intestinal inflammation [171–173]. Blocking the attachment of S1P and its ligands can effectively decrease the migration of lymphocytes to the location of intestinal inflammation, thus relieving the symptoms of IBD [174]. Considering its influence on autophagy, S1P may also have a regulatory function in autophagy within the context of IBD disease. The focus of our team has been on studying the involvement of autotaxin (ATX), a nucleotide pyrophosphate/phosphodiesterases family member with lysophospholipase D activity, in IBD [175]. It has been confirmed that ATX expression increases in acute and chronic colitis, while the inhibition of ATX activity can alleviate intestinal inflammation [176, 177]. From our recent studies, ATX can inhibit autophagy by activating the mTOR pathway or inhibiting the AMPK pathway, disrupting intestinal epithelial barrier function during colitis [178, 179]. Notably, ATX is reported to act as an S1P synthase that degrades sphingosylphosphorylcholine to S1P [180]. Hence, we boldly propose the hypothesis that ATX activates the mTOR pathway or inhibits the AMPK pathway by regulating S1P/S1PR expression, thereby inhibiting autophagy and disrupting the intestinal epithelial barrier, ultimately fostering the progression of intestinal inflammation (Fig. 4). Overall, the role of S1P in regulating autophagy in digestive system diseases needs further exploration to optimize the development of targeted therapy.

### Other diseases

In addition to the diseases mentioned above, S1P has also been demonstrated to control autophagy in other conditions, such as rheumatoid arthritis, myopathy, and renal fibrosis. In the mouse model of rheumatoid arthritis, inflammation-induced autophagy regulates the generation of S1P in LECs, which leads to the restriction of Th17 egression from LNs, thereby mitigating disease progression [123]. The results highlight the autophagy pathway as a crucial regulator for immunomodulatory functions under inflammatory conditions. SphK1/S1P/S1PR2 signaling axis is activated in dexamethasone-induced muscle atrophy, while SphK1/S1P/S1PR3 signaling axis is activated in TNF-α induced muscle atrophy. The activation of both S1P signaling pathways triggers autophagy in skeletal muscle cells, resulting in the breakdown of skeletal muscle protein and the advancement of disease progression [181, 182]. However, activation of SphK1, which converts sphingosine into endogenous S1P, has a renoprotective effect via induction of autophagy during renal fibrosis [183]. In summary, the regulation of autophagy by S1P exhibits different effects in different diseases. Therefore, effective targeted treatment methods should be adopted for specific pathogenesis.

## Conclusions and future perspectives

As a critical cellular process, autophagy has contradictory functions in regulating cell fate: maintaining cell homeostasis and inducing cell death [6]. It is a complex process that is interconnected with multiple cellular pathways. Although a moderate degree of autophagy can enhance cell survival, excessive autophagy can result in cell death. Autophagy regulation may be an appropriate treatment strategy for a range of ailments. Nevertheless, given the intricacy of the autophagy mechanism and the contradiction of autophagy function, additional measures beyond autophagy modulation are necessary to attain the intended therapeutic outcomes. Caution should be exercised when using autophagy-modulating drugs. A comprehensive treatment strategy that considers the interconnected nature of autophagy and combines multiple therapeutic approaches may be necessary for optimal results. Therefore, it is essential to comprehend the mechanisms underlying autophagy regulation, which can be modulated by diverse biological signaling molecules, such as sphingolipids.

Sphingolipids, a varied group of lipid molecules, can function as secondary messengers during cellular processes. Different sphingolipid metabolites can exert contrasting impacts on cellular processes, for example, the ceramide/S1P rheostat. Ceramide has been comprehensively reviewed before as primarily associated with lethal autophagy regulation [6]. This review mainly describes the regulation of S1P in the process of the autophagic machinery. Although S1P-mediated autophagy sometimes leads to disease progression, S1P-dependent autophagy is mainly related to cell survival in most cases. Elevated S1P levels and impaired autophagy are observed in IBD patients [184, 185]. Based on previous studies, we suspect that S1P inhibits autophagy through the mTOR pathway activation or the AMPK pathway inhibition, leading to worsening inflammation in IBD. This viewpoint requires further experimental confirmation to provide new ideas for the treatment of IBD. Targeting S1P signaling could potentially be a therapeutic strategy for autophagy-related diseases. Several small molecule inhibitors and agonists targeting S1P receptors have been developed and are currently undergoing clinical trials in various diseases (Table 2). Additionally, combining S1P-targeting therapies with other autophagy-inducing treatments could provide a synergistic effect in treating these diseases.

**Table 2:** Novel S1P modulators in clinical studies.

S1P modulator	Target	Mode of delivery	Indication	Developmental phase	Reference
Ozanimod (RPC-1063)	S1PR1, S1PR5	Oral	RRMS	FDA and EMA approved in 2020	[186]
			IBD	Phase II completed and phase III recruiting for CD	[187]
				Phase III completed for UC	[188, 189]
			COVID-19 pneumonia	Phase II recruiting	
			Liver diseases-Digestive system diseases	Phase I	
Etrasimod (APD-334)	S1PR1, S1PR4, S1PR5	Oral	IBD	Phase II/III recruiting for CD	
				Phase II completed and phase III recruiting for UC	[190]
			Eosinophilic esophagitis	Phase II	
			Primary biliary cholangitis	Phase II	
			Alopecia areata	Phase II	
			Atopic dermatitis	Phase II	
			Pyoderma gangrenosum	Phase II	
			Autoimmune diseases	Phase I	
			Rheumatoid arthritis		
Amiselimod (MT-1303)	S1PR1, S1PR5	Oral	IBD	Phase II completed for CD	[191]
				Phase II for UC	
			RRMS	Phase II completed	[192]
			Plaque psoriasis	Phase II completed	
			SLE	Phase II completed	
Mocravimod (KRP-203)	S1PR1, S1PR3	Oral	UC	Phase II terminated	[193]
			Subacute cutaneous lupus erythematosus	Phase II completed	
			Transplant rejection (for hematological malignancies)	Phase I completed	[194]
Fingolimod (FTY720)	S1PR1, S1PR3, S1PR4, S1PR5	Oral	RRMS and PPMS	FDA and EMA approved for RRMS in 2010	[186]
				Phase III for PPMS	[195]
			Cognition, Brain volume loss	Phase IV	
			Chronic inflammatory demyelinating polyradiculoneuropathy	Phase III completed	[196]
			De novo renal transplantation	Phase III completed	[197]
			Rett’s Syndrome	Phase II completed	[198]
			Amyotrophic lateral sclerosis	Phase II completed	[199]
			Acute demyelinating optic neuritis	Phase II	
			Ischaemic stroke	Phase II completed	[200]
			Intracerebral haemorrhage	Phase II completed	[201]
			Schizophrenia	Phase II completed	
			Uveitis	Phase II	
			Asthma	Phase II completed	[202]
			COVID-19 pneumonia	Phase II	
			High grade glioma	Phase I completed	
			Breast carcinoma	Phase I	
			Anaplastic astrocytoma	Phase I	
			Cerebral edema	Phase I	
			Chemotherapy-induced peripheral neuropathy	Phase I recruiting	
			Renal insufficiency	Phase I	
Siponimod (BAF-312)	S1PR1, S1PR5	Oral	RRMS and active SPMS	FDA approved for RRMS in 2019	[186]
				EMA approved for active SPMS in 2019	
			Intracerebral haemorrhage	Phase II recruiting	
			Hemorrhagic stroke	Phase II	
			Active dermatomyositis	Phase II	
			Polymyositis	Phase II	
			Renal impairment	Phase I	
			Hepatic impairment	Phase I	
Ponesimod (ACT-128800)	S1PR1	Oral	RRMS and active RMS	FDA and EMA approved for RRMS in 2021	[186]
				Phase III for active RMS	
			Plaque psoriasis	Phase II completed	[203]
			Graft rejection	Phase II	
Ceralifimod (ONO-4641)	S1PR1, S1PR5	Oral	RRMS	Phase II completed	[204]
Cenerimod (ACT-334441)	S1PR1	Oral	SLE	Phase II completed	[205]
AKP11	S1PR1	Oral	Psoriasis	Phase II recruiting	
GSK2018682	S1PR1	Oral	RRMS	Phase I completed	
CS-0777	S1PR1	Oral	MS	Phase I completed	
LX-3305 (LX-2931)	S1P lyase inhibitor	Oral	Rheumatoid arthritis	Phase II completed	
Sonepcizumab (LT-1009)	S1P	Intravenous injection	Macular degeneration	Phase II	
			Renal cell carcinoma	Phase II completed	[206]

*MS* multiple sclerosis, *RRMS* relapsing remitting MS, *EMA* European Medicine Agency, *FDA* food and drug administration, *IBD* inflammatory bowel disease, *CD* Crohn’s disease, *UC* ulcerative colitis, *SLE* systemic lupus erythematosus, *PPMS* primary progressive multiple sclerosis, *RMS* relapsing MS.

There is still a lot to be discovered concerning the involvement of S1P in autophagy regulation. For example, the more detailed molecular mechanisms by which S1P modulates autophagy and its downstream effects remain mostly unidentified. Additionally, the role of S1P in regulating other forms of selective autophagy, such as lipophagy and glycophagy, deserves deeper understanding. In spite of these challenges, S1P presents an exciting opportunity for further investigation into the regulation of autophagy. Considering the crucial role of autophagy in cellular health and disorders, the significance of targeting S1P-mediated autophagy regulation for potential therapeutic purposes cannot be emphasized enough.
